# Supplementary figures and images for: Identification and experimental verification of necroptosis-related prognostic gene signature and characterization of tumor immune infiltration in lung squamous cell carcinoma
Source: PeerJ. 2025 Oct 29;13:e20260. doi: 10.7717/peerj.20260 (PMC12579482; doi:10.7717/peerj.20260)

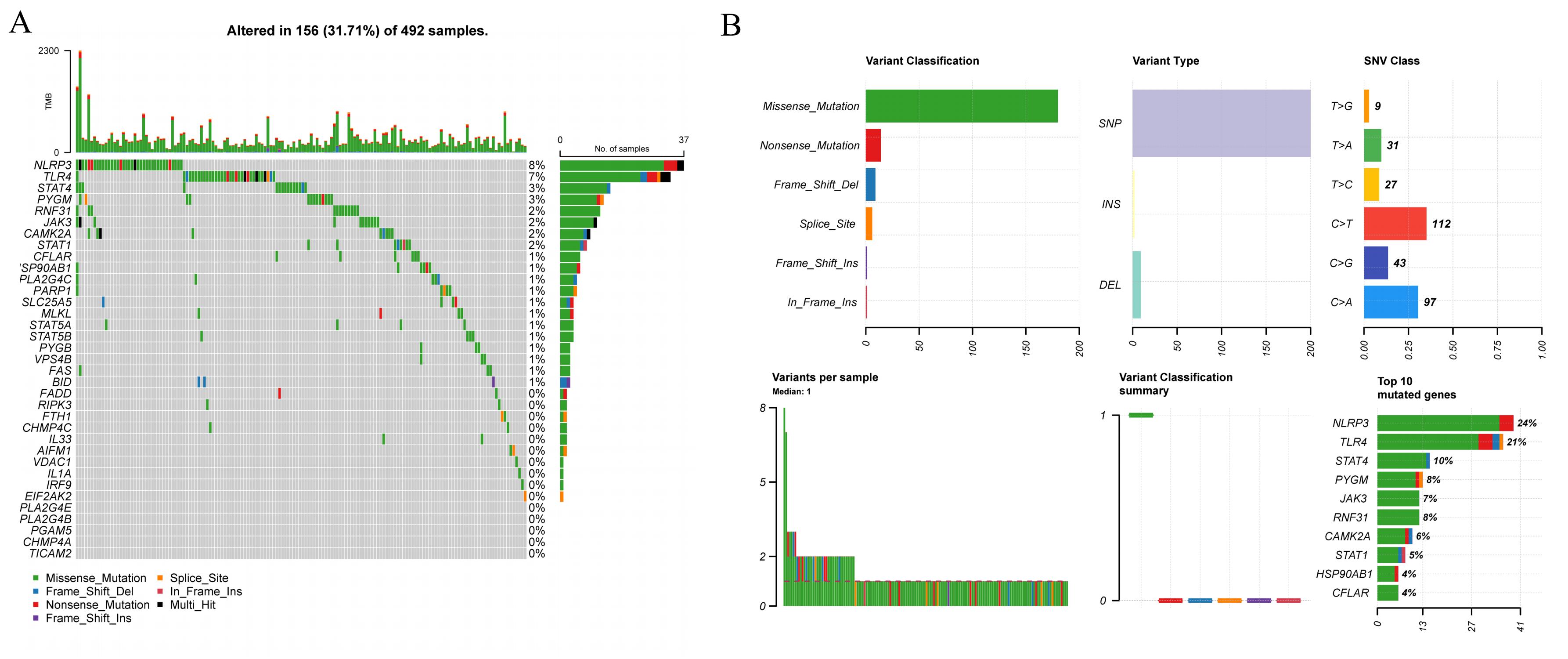

Supplement: Supplemental Information 1 [file peerj-13-20260-s001.jpg]

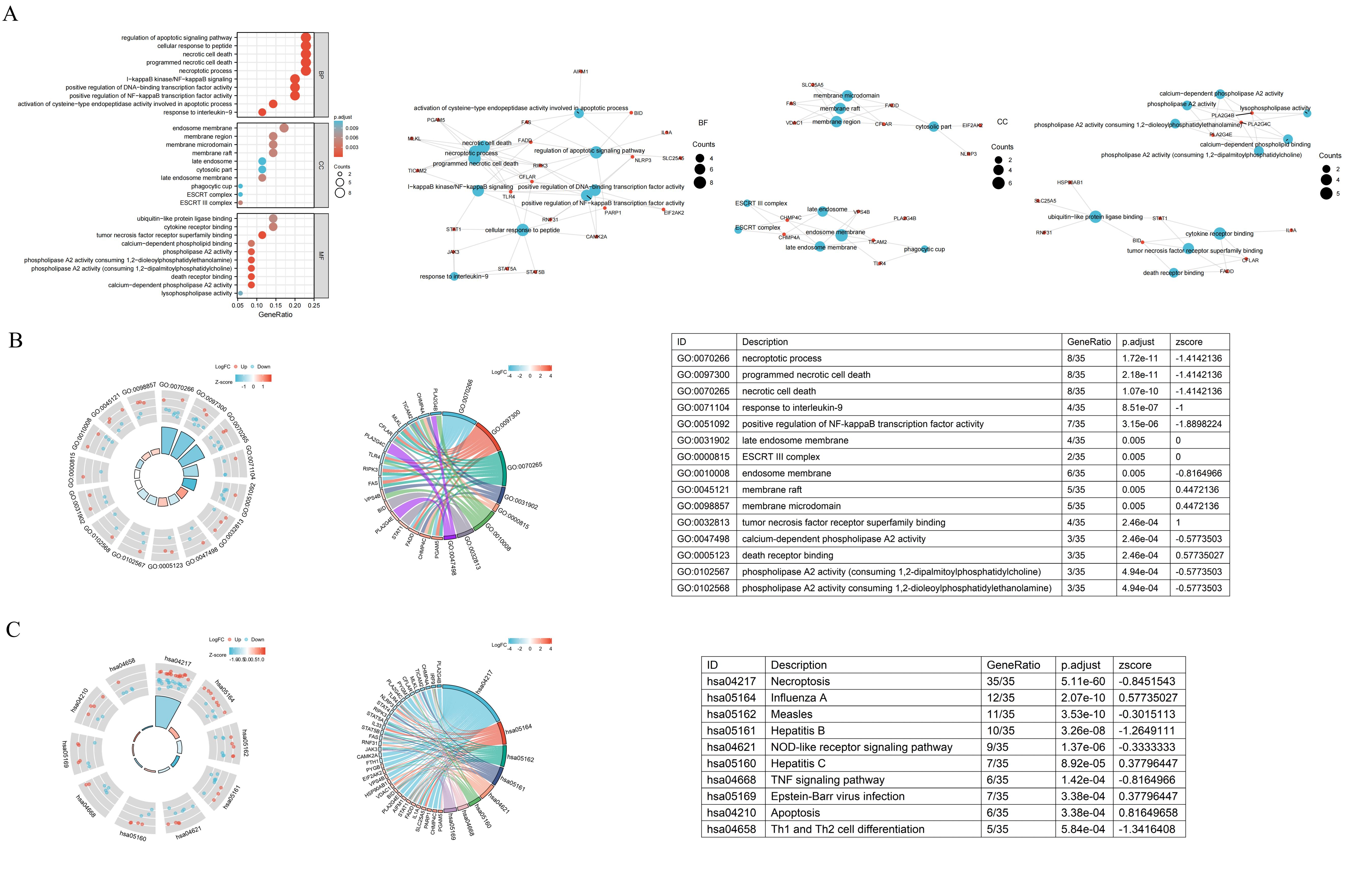

Supplement: Supplemental Information 2 [file peerj-13-20260-s002.jpg]
