# Supplementary material for: Identification and experimental verification of necroptosis-related prognostic gene signature and characterization of tumor immune infiltration in lung squamous cell carcinoma
Source: PeerJ. 2025 Oct 29;13:e20260. doi: 10.7717/peerj.20260 (PMC12579482; doi:10.7717/peerj.20260)
Supplement: Supplemental Information 3 [file peerj-13-20260-s003.docx]

| Table S1 159 NRGs from KEGG in LUSC. | | | | |
| --- | --- | --- | --- | --- |
| gene | VDAC3 | CHMP3 | IFNA16 | PARP1 |
| TNF | GLUD2 | RNF103-CHMP3 | IFNA17 | BID |
| TNFRSF1A | GLUD1 | CHMP4B | IFNA21 | BAX |
| TRADD | GLUL | CHMP4A | IFNB1 | AIFM1 |
| TRAF2 | PYGL | CHMP4C | IFNG | H2AX |
| TRAF5 | PYGM | CHMP6 | IFNAR1 | H2AC20 |
| RIPK1 | PYGB | VPS4B | IFNAR2 | H2AC12 |
| BIRC2 | MAPK8 | VPS4A | IFNGR1 | H2AC1 |
| BIRC3 | MAPK10 | CHMP1B | IFNGR2 | H2AW |
| XIAP | MAPK9 | CHMP1A | JAK1 | H2AB3 |
| RBCK1 | FTH1 | CHMP5 | JAK2 | H2AC8 |
| RNF31 | FTL | CHMP7 | JAK3 | H2AC4 |
| SHARPIN | PLA2G4E | TRPM7 | TYK2 | MACROH2A2 |
| SPATA2L | PLA2G4A | IL1A | STAT1 | MACROH2A1 |
| SPATA2 | JMJD7-PLA2G4B | IL33 | STAT2 | H2AC19 |
| CYLD | PLA2G4B | HMGB1 | STAT3 | H2AJ |
| FADD | PLA2G4C | TNFSF10 | STAT4 | H2AB1 |
| CASP8 | PLA2G4D | TNFRSF10A | STAT5A | H2AC17 |
| CFLAR | PLA2G4F | TNFRSF10B | STAT5B | H2AC18 |
| RIPK3 | ALOX15 | FASLG | STAT6 | H2AC11 |
| CYBB | CAPN1 | FAS | IRF9 | H2AC21 |
| CAMK2A | CAPN2 | FAF1 | EIF2AK2 | H2AZ2 |
| CAMK2D | SMPD1 | IFNA1 | TLR4 | H2AC7 |
| CAMK2B | MLKL | IFNA2 | TICAM2 | H2AZ1 |
| CAMK2G | PGAM5 | IFNA4 | TICAM1 | H2AC15 |
| SLC25A4 | DNM1L | IFNA5 | TLR3 | H2AC6 |
| SLC25A5 | NLRP3 | IFNA6 | ZBP1 | H2AC13 |
| SLC25A6 | PYCARD | IFNA7 | USP21 | H2AC14 |
| SLC25A31 | CASP1 | IFNA8 | SQSTM1 | H2AC16 |
| PPID | IL1B | IFNA10 | HSP90AA1 | H2AB2 |
| VDAC1 | CHMP2A | IFNA13 | HSP90AB1 | PPIA |
| VDAC2 | CHMP2B | IFNA14 | TNFAIP3 | BCL2 |
