# Supplementary material for: Identification and experimental verification of necroptosis-related prognostic gene signature and characterization of tumor immune infiltration in lung squamous cell carcinoma
Source: PeerJ. 2025 Oct 29;13:e20260. doi: 10.7717/peerj.20260 (PMC12579482; doi:10.7717/peerj.20260)
Supplement: Supplemental Information 4 [file peerj-13-20260-s004.docx]

| Table S2 35 NRGs among the DEGs between LUSC and normal samples. | | | | | |
| --- | --- | --- | --- | --- | --- |
| DEGs | logFC | AveExpr | adj.P.Val |  |  |
| RNF31 | -1.123283513 | 4.70546831 | 5.2506E-179 |  |  |
| FADD | 1.488380285 | 3.978142062 | 1.0419E-149 |  |  |
| CFLAR | -1.88759164 | 5.223151513 | 0 |  |  |
| RIPK3 | -1.337782213 | 3.674996847 | 1.6451E-176 |  |  |
| CAMK2A | -1.070862096 | 1.048149134 | 9.9108E-170 |  |  |
| SLC25A5 | 1.52530942 | 9.184679648 | 5.3273E-232 |  |  |
| VDAC1 | 1.076007186 | 7.212745826 | 7.797E-218 |  |  |
| PYGM | -1.871762496 | 1.696321389 | 0 |  |  |
| PYGB | 1.000171139 | 6.534271331 | 2.4249E-108 |  |  |
| FTH1 | -1.06088488 | 10.76371916 | 2.7935E-119 |  |  |
| PLA2G4E | 1.23318038 | 1.236120702 | 7.48375E-67 |  |  |
| PLA2G4B | -4.198858143 | 3.33881748 | 0 |  |  |
| PLA2G4C | -1.603879582 | 2.851158908 | 3.206E-176 |  |  |
| MLKL | -2.379626348 | 4.495152332 | 1.6567E-302 |  |  |
| PGAM5 | 2.252497961 | 4.904173766 | 0 |  |  |
| NLRP3 | -1.440023363 | 2.417997427 | 2.1161E-129 |  |  |
| CHMP4A | -2.888897495 | 4.295674639 | 0 |  |  |
| CHMP4C | 2.178792558 | 4.134913 | 1.5849E-224 |  |  |
| VPS4B | 1.016755147 | 5.425305994 | 1.5563E-148 |  |  |
| IL1A | 1.517836699 | 2.484450265 | 1.59583E-50 |  |  |
| IL33 | -1.296407063 | 4.801059702 | 5.65656E-57 |  |  |
| FAS | -1.15426403 | 4.836979627 | 2.68857E-95 |  |  |
| JAK3 | -1.091457751 | 4.239284893 | 8.46165E-64 |  |  |
| STAT1 | 1.360949778 | 6.770972635 | 1.9913E-132 |  |  |
| STAT4 | -1.367902546 | 2.92060212 | 3.5862E-119 |  |  |
| STAT5A | -1.324973722 | 4.787256429 | 2.0467E-178 |  |  |
| STAT5B | -1.215573895 | 5.635324174 | 1.6546E-222 |  |  |
| IRF9 | -3.247688702 | 4.688091295 | 4.808E-300 |  |  |
| EIF2AK2 | 1.008047846 | 4.492593626 | 6.1949E-147 |  |  |
| TLR4 | -1.600018004 | 3.972154612 | 3.2884E-147 |  |  |
| TICAM2 | -2.742205535 | 1.755043582 | 0 |  |  |
| HSP90AB1 | 1.039014991 | 10.29713513 | 8.19E-136 |  |  |
| PARP1 | 1.993066539 | 5.844161148 | 0 |  |  |
| BID | 1.099228129 | 4.929029269 | 2.1126E-171 |  |  |
| AIFM1 | 1.269001537 | 4.869711901 | 2.2812E-222 |  |  |
