# Supplementary material for: Identification and experimental verification of necroptosis-related prognostic gene signature and characterization of tumor immune infiltration in lung squamous cell carcinoma
Source: PeerJ. 2025 Oct 29;13:e20260. doi: 10.7717/peerj.20260 (PMC12579482; doi:10.7717/peerj.20260)
Supplement: Supplemental Information 5 [file peerj-13-20260-s005.docx]

**Table S3**, Clinical characteristics of patients with LUSC.

| Characteristic | No.of patients(%) |
| --- | --- |
| **n** | **21** |
| **Age, n (%)** |  |
| ≤55 | 4(19%) |
| >55 | 17 (81%) |
| **Gender, n (%)** |  |
| Male | 8 (38.1%) |
| Female | 13 (61.9%) |
| **Tumor size, n (%)** |  |
| ≦5cm | 10(47.6%) |
| >5cm | 11(52.4%) |
| **Smoking, n (%)** |  |
| No | 7 (33.3%) |
| Yes | 14 (66.7%) |
| **Differentiation, n (%)** |  |
| well | 4(19%) |
| Moderate | 11(52.4%) |
| Poor | 6 (28.6%) |
| **Lymph node metastasis, n (%)** |  |
| No | 9 (42.9%) |
| Yes | 12 (57.1%) |
| **TNM stage, n (%)** |  |
| I-II | 8(38.1%) |
| III-IV | 13 (61.9%) |
